# Dual-Layer PDMS/Polysulfone Composite Membranes Incorporating Cu-MOF-74 for Enhanced CO_2_ Capture Performance

**DOI:** 10.3390/polym18111303

**Published:** 2026-05-26

**Authors:** Shoaib Ahsan, Muhammad Ahsan, Tayyaba Noor, Sarah Farrukh, Subhan Ali

**Affiliations:** School of Chemical and Materials Engineering (SCME), National University of Sciences and Technology (NUST), Sector H-12, Islamabad 44000, Pakistan

**Keywords:** mixed-matrix membranes, metal–organic frameworks, gas separation, CO_2_ capture, composite membranes

## Abstract

Polymeric membranes are widely investigated for CO_2_ separation; however, their performance is often limited by the permeability–selectivity trade-off. Incorporating metal–organic frameworks (MOFs) and designing composite membrane architectures are promising strategies to overcome these limitations. This study aims to evaluate the effect of incorporating MOF-74 (Cu and Ni variants) into a polydimethylsiloxane (PDMS) selective layer supported on a polysulfone (PSF) membrane for enhanced CO_2_/N_2_ separation performance. Dual-layer PDMS/PSF composite membranes were fabricated via phase inversion for the PSF support, followed by solution casting of the PDMS/MOF layer. The developed membrane architecture introduces a synergistic design that combines the mechanical robustness of PSF with the selective transport capability of PDMS and the strong CO_2_ affinity of MOF-74, offering an effective strategy for improving gas separation efficiency. Gas permeation performance was assessed using single-gas CO_2_ and N_2_ measurements at feed pressures of 2–5 bar. The incorporation of MOF-74 improved CO_2_ transport properties, with the 1 wt.% Cu-MOF-74 composite membrane achieving a CO_2_ permeance of 912.5 GPU and a CO_2_/N_2_ ideal selectivity of 94.75. The dual-layer configuration significantly enhanced permeance compared with unsupported mixed-matrix membranes while maintaining selectivity. Additionally, the composite membranes exhibited improved mechanical strength due to the PSF support layer. The findings demonstrate that dual-layer PDMS/PSF composite membranes incorporating MOF-74 provide a promising proof-of-concept approach for improving CO_2_ separation performance. Further studies involving mixed-gas testing and long-term stability are required to assess their practical applicability.

## 1. Introduction

Rising carbon dioxide emissions remain a major global concern because they contribute to global warming, climate change, and increasingly unstable weather patterns. According to the International Energy Agency, global energy-related CO_2_ emissions increased by 0.9% in 2022 to more than 36.8 Gt [1]. Conventional CO_2_ capture methods such as cryogenic distillation, amine-based absorption, and adsorption on solid sorbents are effective, but they often suffer from high operating cost, corrosion or solvent-related concerns, and energy-intensive regeneration [2,3,4]. Among conventional CO_2_ capture technologies, amine-based absorption remains the most widely implemented at an industrial scale due to its high capture efficiency, established operational framework, and adaptability to post-combustion gas streams. However, its high energy demand for solvent regeneration and degradation, along with associated environmental concerns, continue to motivate the search for more sustainable alternatives, such as membrane-based systems.

Membrane-based gas separation has attracted considerable interest as a potentially more energy-efficient and modular approach for CO_2_ capture [4,5,6,7]. Various polymeric materials, including cellulose acetate (CA), polyethersulfone (PES), polysulfone (PSF), cellulose triacetate (CTA), polyetherimide (PEI), polycarbonate (PC), polyimide (PI), polydimethylsiloxane (PDMS), and poly(phenylene oxide) (PPO), have been used in gas-separation membrane development [4,5,6,8]. Inorganic membranes, such as silica- and ceramic-based systems, generally offer superior intrinsic separation performance because of their high thermal stability, chemical resistance, and selectivity. However, their fabrication is often more complex and costly than that of polymeric membranes [9,10]. In comparison, polymeric membranes are generally more promising for large-scale CO_2_ capture because they are more economically viable, easier to process, and more scalable. However, they can suffer from limitations such as plasticization, physical aging, and the permeability–selectivity trade-off [4,11,12]. Therefore, mixed-matrix membranes have emerged as a promising hybrid strategy, combining the processability and scalability of polymers with the enhanced separation characteristics of inorganic fillers [13,14,15].

**Figure 1:**
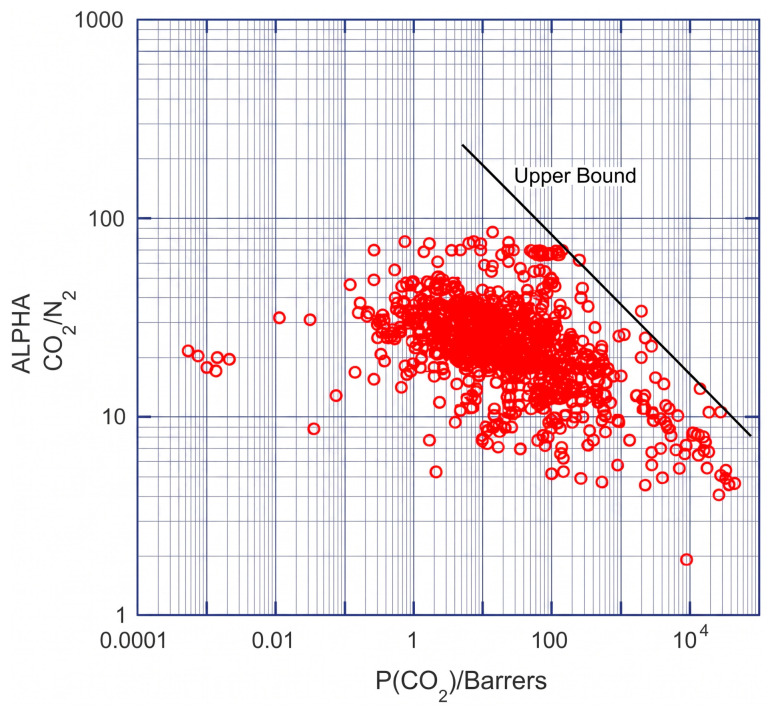
Robeson upper bound (2008) for the CO_2_/N_2_ gas pair, adapted from Robeson [12].

The permeability–selectivity trade-off is a central challenge in polymeric gas-separation membranes and is commonly represented by the Robeson upper bound (Figure 1). This upper-bound relationship was first reported in 1991 and later revised in 2008 [11,12]. The Robeson upper bound provides a useful reference for understanding the general performance limitation of polymer-based membranes, where increased gas permeability is often accompanied by reduced selectivity. In this study, the MOF-74@PDMS/PSF composite membrane was designed to improve this balance by enhancing CO_2_ transport while maintaining CO_2_/N_2_ separation performance.

Mixed-matrix membranes combine polymer matrices with inorganic fillers to improve membrane-based gas separation. Among the available fillers, metal–organic frameworks (MOFs) are particularly attractive because of their high surface area, tunable pore structure, and chemically tailorable frameworks [4,13,14,16]. Recent studies have reported substantial improvements in CO_2_ transport and CO_2_/N_2_ separation performance upon incorporation of MOF fillers into polymer matrices. For example, ZIF-8-based mixed-matrix membranes have demonstrated enhanced CO_2_ permeability due to their porous crystalline structure, while UiO-66-incorporated systems have shown improved selectivity through better polymer–filler compatibility. Similarly, MOF-74 derivatives have attracted attention for their open metal sites and strong affinity for CO_2_, making them particularly suitable for carbon capture applications [17,18,19,20,21,22,23].

However, non-ideal dispersion or poor polymer–filler interfacial compatibility can compromise membrane performance, prompting researchers to explore composite membrane architectures that combine layers with different functions and properties [8,24,25,26]. These composite membranes have shown great promise for efficient CO_2_/N_2_ separation, with several studies achieving optimized CO_2_ permeance and selectivity [8,27,28,29,30]. Despite these advances, challenges remain in balancing high permeance, selectivity, and long-term mechanical stability within scalable membrane configurations. In the present study, a dual-layer PDMS/polysulfone (PSF) membrane architecture was selected in place of a conventional single-layer mixed-matrix membrane to address the trade-off between mechanical stability and separation performance [5,8,25]. In this research, the PSF layer serves as a robust, cost-effective support, providing the mechanical strength to withstand operating pressures without compromising structural integrity.

Meanwhile, the PDMS top layer functions as a thin, selective layer with high gas permeability, into which MOF fillers are incorporated to enhance CO_2_ transport and CO_2_/N_2_ separation performance [5,18,31]. For example, an amorphous metakaolin-filled polyvinylamine composite membrane achieved a CO_2_ permeance of 188 GPU and a CO_2_/N_2_ selectivity of 83.8 under mixed-gas conditions [30]. Similarly, a PEBAX composite membrane containing aminated partially reduced graphene oxide reached a CO_2_ permeability of 47.5 Barrer and a CO_2_/N_2_ selectivity of 105.6 at the optimum filler loading [29]. Other studies incorporated functionalized ZIF-8 and metal–organic framework nanosheets into composite membranes, thereby enhancing CO_2_ permeation properties and facilitating efficient CO_2_/N_2_ separation [27,28]. MOF-74 materials are widely reported to exhibit strong CO_2_ adsorption affinity due to their open metal sites and accessible porous structure.

This study develops and evaluates dual-layer PDMS/PSF composite membranes incorporating Cu-MOF-74 and Ni-MOF-74 for CO_2_/N_2_ separation. These MOF-74 variants were selected because their open metal sites interact strongly with CO_2_, while changes in the metal center may influence gas transport behavior [32,33]. The main objective is to examine how Cu-MOF-74 and Ni-MOF-74 affect CO_2_ permeation, CO_2_/N_2_ selectivity, and membrane mechanical strength when incorporated into a PDMS selective layer supported by porous PSF. The novelty of this work lies in the direct comparison of Cu- and Ni-based MOF-74 within the same dual-layer PDMS/PSF membrane architecture, rather than in a conventional single-layer mixed-matrix membrane. By combining the CO_2_ affinity of MOF-74, the gas permeability of PDMS, and the mechanical support of PSF, this study proposes a composite membrane design to improve CO_2_ separation performance while maintaining structural stability.

## 2. Materials and Methods

### 2.1. Materials

Polydimethylsiloxane (PDMS, silicone elastomer, analytical grade) was used as the polymer for the selective layer and was obtained from Sigma-Aldrich (St. Louis, MO, USA). Toluene (ACS reagent grade, 99.7% purity), also purchased from Sigma-Aldrich, served as the solvent. The metal–organic frameworks (MOFs), Cu-MOF-74 and Ni-MOF-74, were provided by a collaborating research group within our institution. These MOF-74 variants exhibited micrometer-scale particle morphology, with a particle size range of 1–10 µm, and were selected for their open metal sites and strong affinity for CO_2_ molecules. Polysulfone (PSF, Mn = 22,000 g·mol^−1^, bead form), obtained from Sigma-Aldrich, was used to fabricate the porous support layer. N-methyl-2-pyrrolidone (NMP), acquired from RCI Labscan Limited (Bangkok, Thailand), was used as the solvent for the casting solution. Deionized water obtained from HAT Enterprises (Islamabad, Pakistan) was used as the non-solvent in the phase inversion process. All chemicals and solvents were utilized in their original state, without any additional purification or treatment. High-purity CO_2_ (99.99%) and N_2_ (99.99%) were obtained from Paradise Gases (Islamabad, Pakistan) and used in the gas permeation tests.

### 2.2. Synthesis and Formulations of PSF Porous Membranes

**Figure 2:**
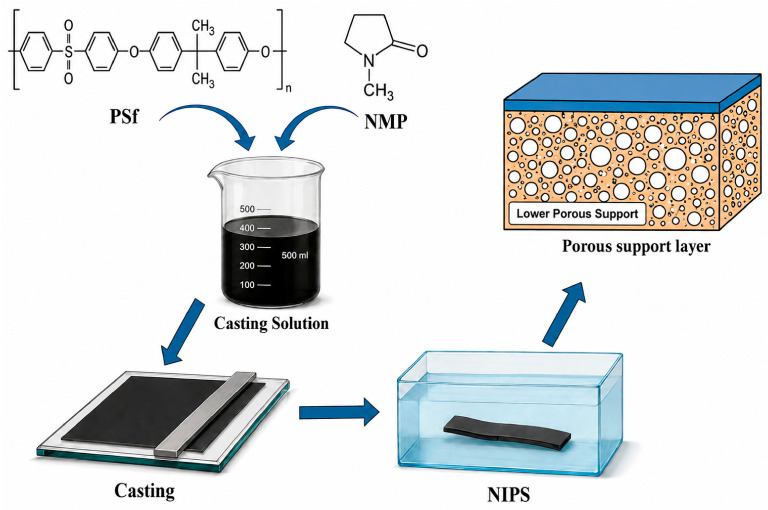
Fabrication of PSF porous support membrane.

The porous PSF support membranes were fabricated from polysulfone solutions prepared in *N*-methyl-2-pyrrolidone (NMP) at different polymer concentrations. PSF was dissolved in NMP to prepare solutions containing 10, 20, and 25% (*w*/*v*) polymer by adding 1.0, 2.0, and 2.5 g of PSF, respectively, to 10 mL of NMP in glass vials. The mixtures were stirred for 12–18 h to ensure complete dissolution. The PSF solution was then cast onto a glass plate and exposed to air for 5–10 s before immersion in deionized water to induce non-solvent-induced phase separation (NIPS). The cast films were immersed in deionized water for at least 300 s. After membrane formation, the membranes were removed from the water bath and dried at 70 °C for approximately 40–45 min. The porous membranes were then peeled from the glass plates and stored in sealed bags to prevent contamination. Figure 2 shows a schematic of this procedure.

### 2.3. Fabrication of MOF-74@PDMS Selective Layers on PSF Supports

**Figure 3:**
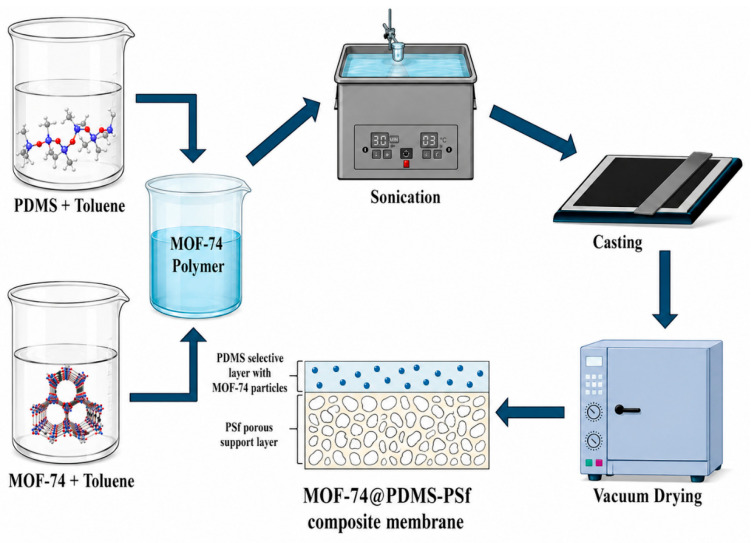
Fabrication of MOF-74@PDMS/PSF composite membrane.

PSF-supported PDMS/MOF dual-layer composite membranes were prepared as follows. First, dried PSF porous support membranes were securely attached to 10 cm × 10 cm glass plates using aluminum tape. Next, two separate mixtures were prepared. The first mixture contained 4 g of PDMS (40% *w*/*v*) dissolved in 10 mL of toluene, stirred at 300 rpm for 2 h to ensure complete dissolution, with an elastomer-to-curing agent ratio of 10:1. This concentration was selected to achieve a suitable viscosity for uniform coating and defect-free film formation, consistent with reported membrane fabrication practices. Separately, MOF-74 was dispersed in toluene at the desired loading (0.5–1.0 wt.% relative to polymer) by magnetic stirring for 1 h. The selected filler range was chosen to balance enhanced gas transport pathways with minimal agglomeration, based on preliminary optimization and prior literature findings. The suspension was subsequently ultrasonicated at 550 W and 20 kHz for 1 h to improve dispersion homogeneity. The resulting MOF dispersion was then combined with the PDMS solution and subjected to a further 1 h of sonication in order to promote uniform filler distribution and suppress particle agglomeration prior to casting. Finally, the mixture was degassed for 30 min to remove entrapped air bubbles. The resulting mixture was then cast onto the porous PSF support, and the coated membrane was then placed in an oven at 80 °C for 2–3 h to allow curing and solvent removal. After coating and curing, the thickness of the selective layer was measured independently using a digital micrometer. The membrane formulations prepared in this study, including the PSF supports, PDMS mixed-matrix membranes, and PDMS/PSF composite membranes, are summarized in Table 1. The preparation process for the MOF-74@PDMS/PSF dual-layer composite membranes is further illustrated schematically in Figure 3.

## 3. Characterization and Testing

### 3.1. Gas Permeation Analysis

Single-gas permeation experiments for CO_2_ and N_2_ were conducted using a PHILOS^®^ permeability test system (PHILOS Co., Ltd., Gwangmyeong-si, Gyeonggi-do, South Korea) equipped with a stainless-steel gas permeation cell. All measurements were performed at 25 °C (298 K) at feed pressures of 2–5 bar for pure CO_2_ and pure N_2_. The PSF support membranes were prepared as 10 cm × 10 cm sheets; however, only the circular area exposed within the permeation module was used for gas permeation measurements, corresponding to an effective membrane area of 8.54 cm^2^. The effective membrane area was 8.54 cm^2^, and the composite membranes used in the permeation experiments had an average PDMS-selective-layer thickness of approximately 60–80 μm, as determined using a digital micrometer. It should be noted that SEM cross-sectional images represent localized regions at specific magnifications and may not directly reflect the overall membrane thickness. Therefore, apparent differences between SEM observations and measured thickness arise from imaging scale and sample sectioning, rather than actual structural inconsistency.

The permeate gas flow rate was measured using a glass bubble flowmeter connected to the permeate outlet of the cell. Membrane performance was evaluated in terms of gas permeance and ideal selectivity. The ideal selectivity of gas A over gas B was calculated as the ratio of their single-gas permeances:

(1) $$\alpha_{A/B}=\frac{\Pi_{A}}{\Pi_{B}}$$

where $\alpha_{A/B}$ is the ideal selectivity, and $\Pi_{A}$ and $\Pi_{B}$ are the permeances of gases A and B, respectively [5,9]. The permeance of gas A was calculated using:

(2) $$\Pi_{A}=\frac{Q}{A\;\Delta p}$$

where $\Pi_{A}$ is the gas permeance, Q is the volumetric gas flow rate, A is the effective membrane area, and Δp is the transmembrane partial-pressure difference of gas A. Gas permeance was reported in GPU [5,9,34].

### 3.2. Fourier Transform Infrared (FTIR) Spectroscopy

FTIR spectra were recorded using a PerkinElmer Spectrum 100 FT-IR spectrometer (PerkinElmer, Inc., Waltham, MA, USA) over the wavenumber range of 4000–400 cm^−1^ in attenuated total reflectance (ATR) mode using a diamond crystal accessory. FTIR spectra were recorded for Cu-MOF-74, Ni-MOF-74, pristine PDMS, the porous PSF support membrane, PDMS-based mixed-matrix membranes, and PDMS/PSF composite membranes. The spectra were used to identify characteristic functional groups and to compare the spectral features of the pristine materials with those of the fabricated membranes.

### 3.3. Scanning Electron Microscopy (SEM)

SEM analysis was performed using a JEOL JSM-6490LA analytical low-vacuum scanning electron microscope (JEOL Ltd., Akishima, Tokyo, Japan). Cross-sectional samples were prepared by fracturing the membranes in liquid nitrogen. Micrographs were recorded at magnifications of 1000×, 2500×, 15,000×, and 30,000×. SEM was used to examine the surface and cross-sectional morphologies of pristine PDMS, Cu-MOF-74@PDMS and Ni-MOF-74@PDMS mixed-matrix membranes, the porous PSF support membrane, and the Cu-MOF-74@PDMS/PSF and Ni-MOF-74@PDMS/PSF composite membranes. SEM imaging was also performed for the Cu-MOF-74 and Ni-MOF-74 powders. In addition, energy-dispersive X-ray (EDX) analysis was carried out on selected membrane samples to examine the elemental distribution of the MOF phase.

### 3.4. X-Ray Diffraction (XRD)

XRD analysis was performed using a Bruker D2 Phaser diffractometer (Bruker Corporation, Billerica, MA, USA). XRD patterns were recorded using Cu-Kα radiation (λ = 1.5406 Å) at an operating voltage of 30 kV and a current of 10 mA. The analysis was carried out on Cu-MOF-74 and Ni-MOF-74 powders, pristine PDMS, the porous PSF support membrane, and MOF-containing membrane samples to examine their structural features and to assess the effect of MOF incorporation on the polymer matrix.

### 3.5. Ultimate Tensile Strength (UTS)

Tensile testing was performed using a Linkam TST350 tensile testing stage (Linkam Scientific Instruments Ltd., Surrey, UK) in accordance with ASTM D882-12 (ASTM International, 2012). Membrane specimens were prepared as rectangular strips measuring 26 mm × 10 mm. A 200 N load cell was used, and each sample was tested at least three times to ensure reproducibility. The measurements were conducted on pristine PDMS, PDMS-based mixed-matrix membranes, and PDMS/PSF composite membranes to evaluate the effect of Cu-MOF-74 and Ni-MOF-74 incorporation on membrane mechanical strength.

## 4. Results and Discussion

The MOF-74 materials employed in this study are well-established systems with extensively reported physicochemical properties. Typical BET surface areas and CO_2_ adsorption capacities of MOF-74 materials have been widely reported in the literature. In the present work, the focus is placed on membrane fabrication and gas transport performance; therefore, literature-reported values are used to contextualize the expected adsorption behavior of the filler phase.

### 4.1. Gas Separation Analysis

**Figure 4:**
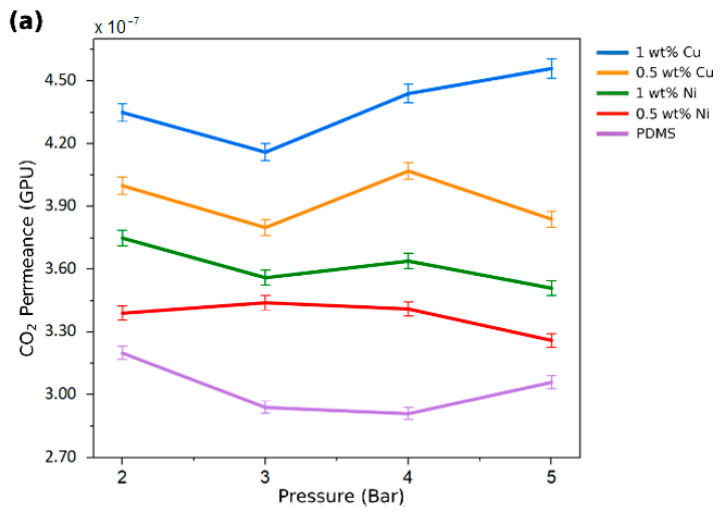

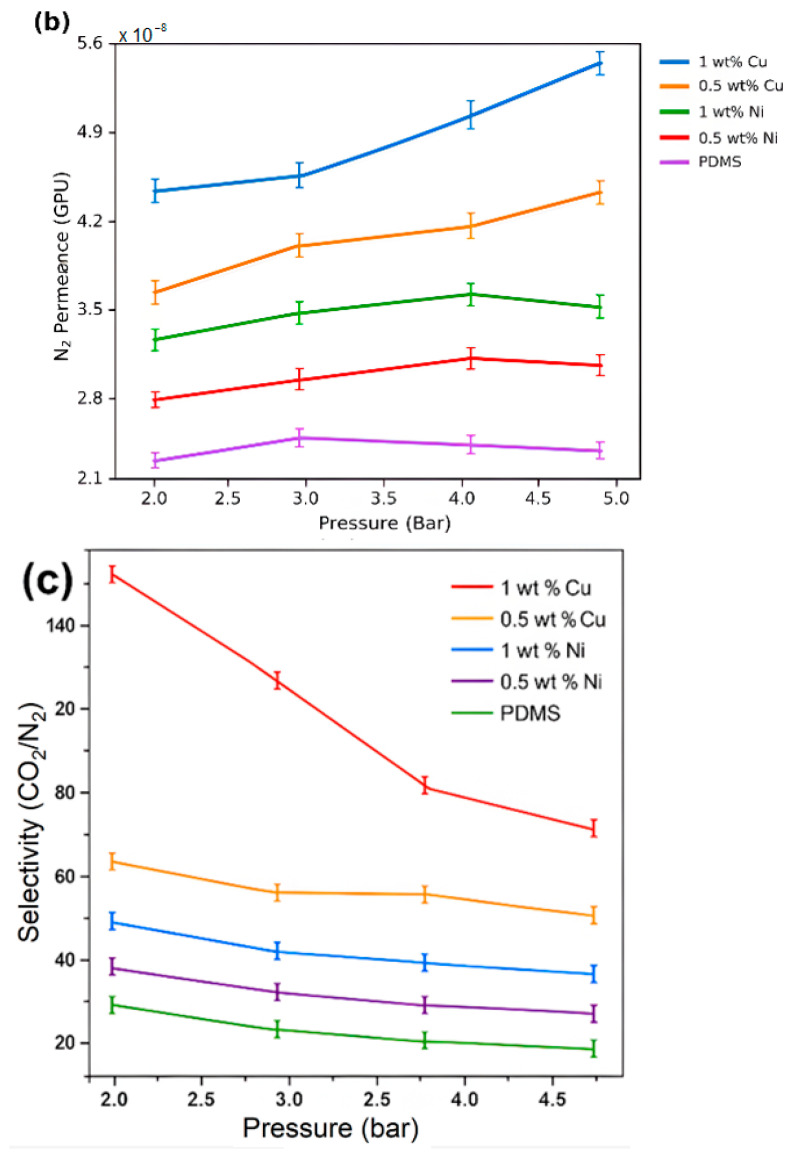
Gas-separation performance of unsupported PDMS-based mixed-matrix membranes incorporating Cu-MOF-74 and Ni-MOF-74: effect of feed pressure on (**a**) CO_2_ permeance, (**b**) N_2_ permeance, and (**c**) ideal CO_2_/N_2_ selectivity.

**Figure 5:**
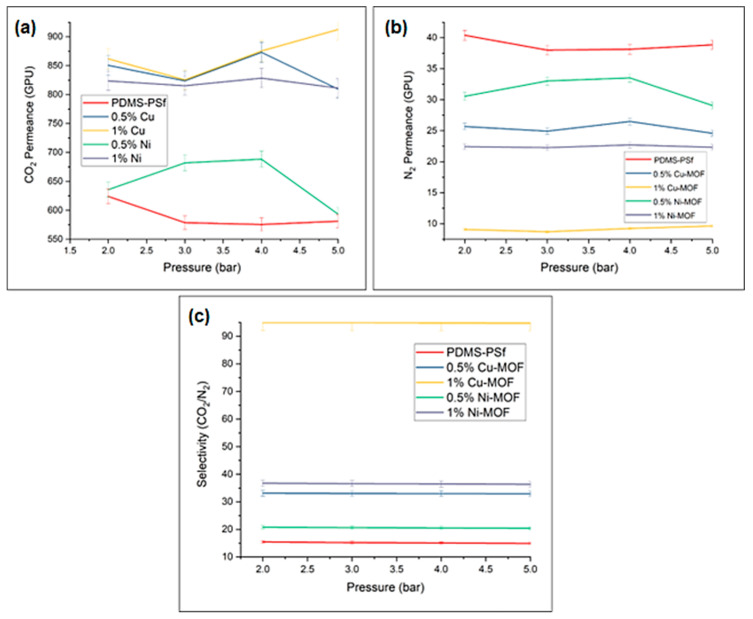
Gas-separation performance of PDMS/PSF composite membranes incorporating Cu-MOF-74 and Ni-MOF-74: effect of feed pressure on (**a**) CO_2_ permeance, (**b**) N_2_ permeance, and (**c**) ideal CO_2_/N_2_ selectivity.

**Figure 6:**
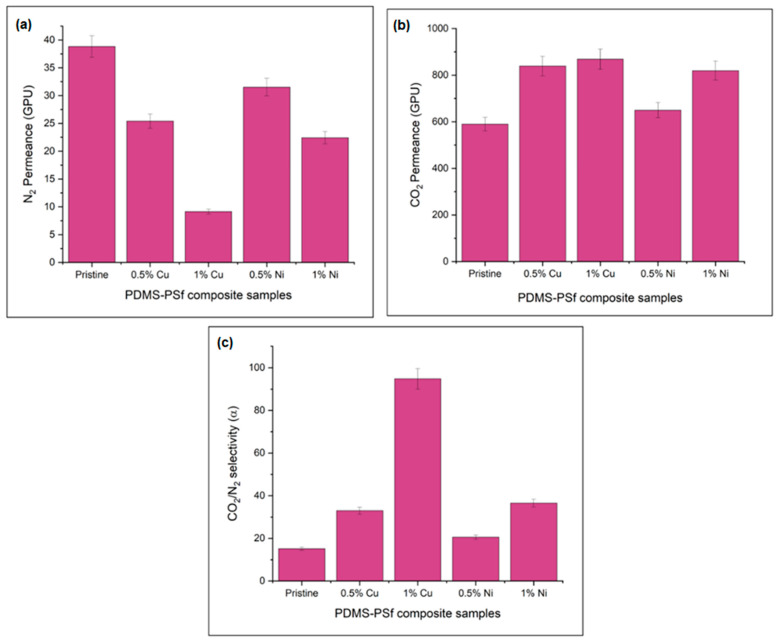
Summary of the gas-separation performance of PDMS/PSF composite membranes containing Cu-MOF-74 and Ni-MOF-74: (**a**) N_2_ permeance, (**b**) CO_2_ permeance, and (**c**) ideal CO_2_/N_2_ selectivity.

The gas-separation performance of the fabricated membranes was evaluated by single-gas permeation of CO_2_ and N_2_ over a feed-pressure range of 2–5 bar. The results for the unsupported PDMS-based mixed-matrix membranes (MMMs) are presented in Figure 4, whereas the corresponding PSF-supported PDMS/MOF dual-layer composite membranes are shown in Figure 5 and Figure 6. Because gas transport in the dense PDMS-based selective layer is governed primarily by the solution–diffusion mechanism, the observed membrane performance reflects the combined effects of gas sorption and diffusion within the polymer/filler phase [5].

For the unsupported PDMS MMMs (Figure 4), incorporation of both Cu-MOF-74 and Ni-MOF-74 increased CO_2_ permeance relative to pristine PDMS, and the 1 wt.% Cu-MOF-74 membrane showed the highest CO_2_ permeance over the investigated pressure range. However, N_2_ permeance also increased with pressure, particularly in the Cu-containing membranes, which resulted in a marked decline in ideal CO_2_/N_2_ selectivity from 2 to 5 bar. These results indicate that although MOF incorporation enhanced CO_2_ transport, the unsupported MMMs remained sensitive to pressure-dependent changes in gas transport. The higher CO_2_ permeance observed for Cu-MOF-74 compared with Ni-MOF-74 can be attributed to the stronger interaction between CO_2_ molecules and the more accessible open Cu^2+^ metal sites, which enhances CO_2_ adsorption and facilitates faster diffusion through adsorption–desorption transport pathways.

In contrast, Ni-MOF-74 exhibits comparatively weaker CO_2_ binding strength, leading to lower facilitated transport contribution. Importantly, although increased permeance often reduces selectivity, selectivity can be maintained or even improved when the increase in CO_2_ permeance is proportionally greater than that in N_2_ permeance. In Cu-MOF-74-containing membranes, the stronger CO_2_ affinity of Cu^2+^-open-metal sites likely promoted preferential CO_2_ adsorption and transport, thereby increasing CO_2_ permeance more than N_2_ permeance. Within the present membrane system, the Cu-containing MMMs outperformed the corresponding Ni-containing MMMs in both CO_2_ permeance and ideal selectivity, suggesting more effective CO_2_-selective transport in the Cu-based system under the studied conditions. This trend is consistent with literature reports on MOF-74 materials, where open metal sites promote preferential CO_2_ sorption; however, the overall membrane performance is also strongly influenced by filler dispersion, polymer–filler interfacial compatibility, and pressure-induced transport effects [24,32].

The relatively low permeance values observed for the unsupported PDMS-based MMMs are primarily due to their dense structure and comparatively large thickness, which impose significant resistance to gas transport. In addition, the absence of a porous support layer further limits effective permeation. This behavior is consistent with the solution–diffusion mechanism and highlights the importance of thin-film composite architectures for achieving higher permeance.

A much greater improvement in the measured separation performance was observed for the dual-layer PDMS/PSF composite membranes (Figure 5 and Figure 6). The pristine PDMS/PSF membrane exhibited the lowest separation performance, whereas all MOF-containing composite membranes showed enhanced CO_2_ permeance and higher ideal CO_2_/N_2_ selectivity. Among the tested composite membranes, the 1 wt.% Cu-MOF-74/PDMS/PSF membrane showed the best overall performance, with a CO_2_ permeance of 912.5 GPU and an ideal CO_2_/N_2_ selectivity of 94.75. The corresponding 1 wt.% Ni-MOF-74 composite membrane also showed relatively high CO_2_ permeance, but its higher N_2_ permeance limited the selectivity to approximately 36. The 0.5 wt.% Cu-MOF-74 and 0.5 wt.% Ni-MOF-74 composite membranes exhibited intermediate performance. Based on the measured permeance-selectivity balance under the present test conditions, the membrane performance followed the order 1 wt.% Cu-MOF-74 > 1 wt.% Ni-MOF-74 > 0.5 wt.% Cu-MOF-74 > 0.5 wt.% Ni-MOF-74 > pristine PDMS/PSF, as summarized in Figure 6.

No obvious plasticization-related decline in membrane performance was observed within the investigated pressure range of 2–5 bar. This stability is further supported by the dual-layer configuration, in which the porous PSF support provides mechanical reinforcement while the dense PDMS layer maintains a consistent transport pathway.

These results suggest that a filler loading of 1 wt.% provided the most favorable balance between enhanced CO_2_ transport and retention of selective membrane structure. At 0.5 wt.% loading, the improvement was evident but remained limited, indicating that the population of accessible CO_2_-selective sites was still insufficient for maximum performance. By contrast, the 1 wt.% Cu-MOF-74 membrane combined high CO_2_ permeance with strongly suppressed N_2_ permeation, suggesting that the filler contributed to preferential CO_2_ transport rather than simply increasing total gas transport. Such behavior is consistent with the presence of accessible adsorption sites in MOF-74, provided that the dispersed phase is well distributed and nonselective interfacial defects are minimized [24,32].

An important difference between the unsupported MMMs and the PSF-supported composite membranes was the pressure dependence of separation performance. In the unsupported MMMs, ideal selectivity decreased substantially with increasing pressure, especially for the 1 wt % Cu-MOF-74 membrane. In contrast, the composite membranes showed only minor pressure-dependent variation in permeance, while the ideal CO_2_/N_2_ selectivity remained nearly constant over the investigated pressure range, particularly for the 1 wt.% Cu-MOF-74/PDMS/PSF membrane. This behavior suggests that the dual-layer architecture provided a more stable transport pathway, with the porous PSF layer serving mainly as a mechanical support and the dense PDMS/MOF top layer governing gas separation. In such thin-film composite systems, the integrity of the selective layer is critical for achieving high permeance without sacrificing selectivity [5,25]. The pressure sensitivity observed in the unsupported MMMs may reflect pressure-dependent changes in gas sorption and free-volume effects within the polymer matrix [35].

The present results are based on single-gas permeation measurements. Accordingly, the reported CO_2_/N_2_ values represent ideal selectivities rather than mixed-gas separation factors. Under mixed-gas conditions, competitive sorption and coupled diffusion can alter the effective transport of both components; therefore, binary CO_2_/N_2_ permeation measurements are required for rigorous assessment under application-relevant conditions [5,36]. To further contextualize the results, the optimized 1 wt.% Cu-MOF-74@PDMS/PSF membrane was benchmarked against reported CO_2_/N_2_ separation membranes, as summarized in Table 2. The optimized membrane showed a favorable balance between CO_2_ permeance and ideal CO_2_/N_2_ selectivity, indicating that the Cu-MOF-74-incorporated PDMS/PSF dual-layer architecture effectively enhances gas transport while maintaining separation performance. This comparison supports the potential of the developed membrane for efficient CO_2_ capture applications.

Although the MOF particles are in the micrometer range, the improved separation performance can be attributed to the low filler loading and the dominant role of the continuous PDMS selective layer in governing gas transport. Under these conditions, the presence of MOF-74 enhances CO_2_ sorption without severely disrupting membrane integrity.

The present study is limited to single-gas permeation measurements, and the reported selectivity values therefore represent ideal selectivities. Under mixed-gas conditions, competitive sorption and coupled diffusion effects may influence separation performance. Accordingly, mixed-gas CO_2_/N_2_ measurements are required for comprehensive evaluation and will be the subject of future investigations.

### 4.2. Fourier Transform Infrared Spectroscopy (FTIR) Analysis

**Figure 7:**
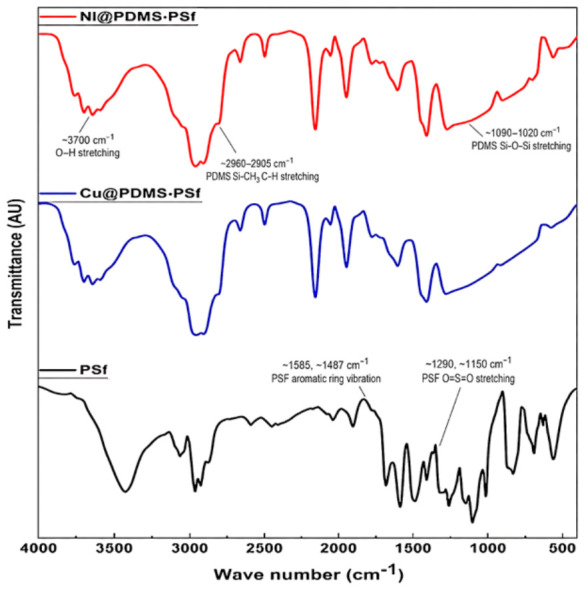
FTIR spectra of Cu-MOF-74@PDMS/PSF, Ni-MOF-74@PDMS/PSF, and the porous PSF support membrane.

FTIR spectroscopy PerkinElmer, Inc. (Waltham, MA, USA) was used to examine the chemical features of the porous PSF support and the final MOF-74-containing PDMS/PSF composite membranes. The FTIR spectra of Cu-MOF-74 and Ni-MOF-74 powders are provided in Figure S1, while the spectra of pristine PDMS and the MOF-74@PDMS mixed-matrix membranes are provided in Figure S2. Figure 7 presents the FTIR spectra of the porous PSF support, Cu-MOF-74@PDMS/PSF, and Ni-MOF-74@PDMS/PSF composite membranes.

The porous PSF support shows the characteristic absorption bands of polysulfone. The bands near 1585 and 1487 cm^−1^ are assigned to aromatic ring vibrations of the PSF backbone. The strong bands near 1290 and 1150 cm^−1^ correspond to asymmetric and symmetric O=S=O stretching vibrations of the sulfone group. The absorption region between 1240 and 1100 cm^−1^ is related to C–O–C stretching vibrations from the ether linkage in PSF [44]. These bands confirm that the PSF support retained its characteristic chemical structure after the NIPS fabrication process.

After coating with the PDMS/MOF selective layer, the Cu-MOF-74@PDMS/PSF and Ni-MOF-74@PDMS/PSF membranes show clear PDMS-related absorptions. The bands near 2960–2905 cm^−1^ are assigned to C–H stretching vibrations of Si–CH_3_ groups [45]. The band near 1260 cm^−1^ corresponds to the Si–CH_3_ deformation [45]. The strong absorption envelope in the 1090–1020 cm^−1^ region is attributed mainly to Si–O–Si stretching of the PDMS backbone. The band near 800 cm^−1^ is assigned to Si–CH_3_ rocking and Si–C-related vibration [44]. The presence of these bands in both composite membranes confirms the formation of the PDMS-based selective layer on the PSF support.

In the high-wavenumber region, the MOF-containing composite membranes exhibit an absorption shoulder around 3700 cm^−1^. This band is assigned to O–H stretching vibrations associated with hydroxyl groups and/or adsorbed moisture related to the MOF-74 phase [33]. This assignment is consistent with the Cu-MOF-74 and Ni-MOF-74 powder spectra shown in Figure S1. The presence of this O–H-related band in the composite membranes supports an additional MOF-related contribution within the PDMS selective layer.

The region between 1600 and 1400 cm^−1^ contains overlapping contributions from PSF and MOF-74. In PSF, this region includes aromatic ring vibrations, particularly near 1585 and 1487 cm^−1^ [44]. In MOF-74, bands in this region are associated with asymmetric and symmetric stretching vibrations of coordinated carboxylate groups from the organic linker [33]. In the composite membranes, these MOF-related bands are not fully resolved because the MOF loading is low and the stronger polymer absorptions dominate the spectra. This overlap is expected for Cu/Ni-MOF-74-loaded PDMS mixed-matrix systems at low filler concentration [46].

The FTIR spectra, therefore, show contributions from all components of the composite membrane structure. The PDMS selective layer is confirmed by the Si–CH_3_, Si–O–Si, and Si–C-related bands. The PSF support is confirmed by the aromatic, sulfone, and ether absorptions. The MOF-74 phase is supported by the O–H stretching band around 3700 cm^−1^ and by the carboxylate-related absorption region between 1600 and 1400 cm^−1^. The PSF bands remain visible in the composite membranes, but their intensity is reduced compared with the bare PSF support because the PDMS/MOF selective layer covers the surface.

Overall, the FTIR spectra support the successful fabrication of Cu-MOF-74@PDMS/PSF and Ni-MOF-74@PDMS/PSF composite membranes. The composite membranes retain the characteristic bands of PSF, show the dominant siloxane fingerprint of PDMS, and display MOF-related contributions in the O–H and carboxylate regions. No major new absorption bands or large peak shifts are observed, indicating that membrane formation mainly occurred through physical incorporation of Cu-MOF-74 and Ni-MOF-74 into the PDMS selective layer rather than through new covalent bonding between the filler and polymer phases.

### 4.3. Scanning Electron Microscopy Analysis

**Figure 8:**
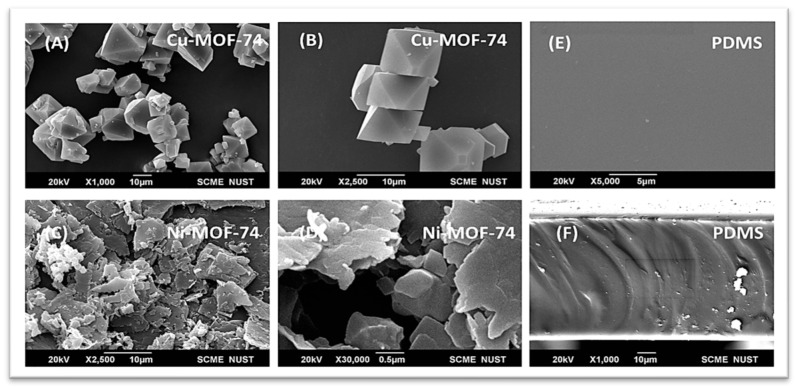
SEM images of (**A**,**B**) Cu-MOF-74 powder, (**C**,**D**) Ni-MOF-74 powder, and (**E**,**F**) the surface and cross-section of pristine PDMS, respectively.

**Figure 9:**
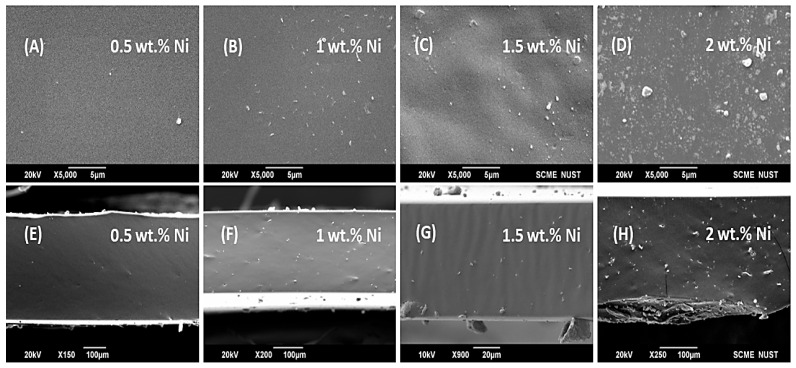
SEM images of Ni-MOF-74@PDMS membranes: (**A**–**D**) surface morphology and (**E**–**H**) cross-sectional morphology of membranes containing 0.5, 1.0, 1.5, and 2.0 wt.% Ni-MOF-74, respectively.

**Figure 10:**
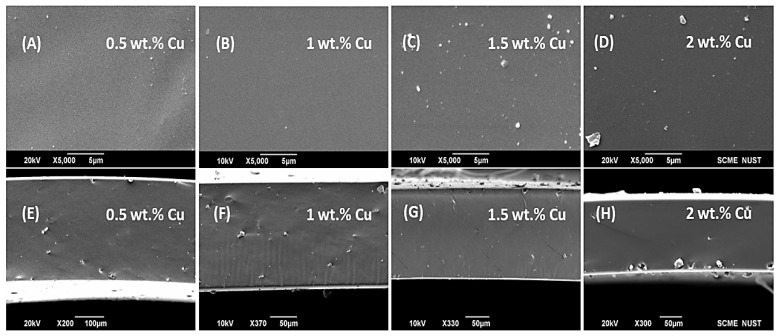
SEM images of Cu-MOF-74@PDMS membranes: (**A**–**D**) surface morphology and (**E**–**H**) cross-sectional morphology of membranes containing 0.5, 1.0, 1.5, and 2.0 wt.% Cu-MOF-74, respectively.

**Figure 11:**
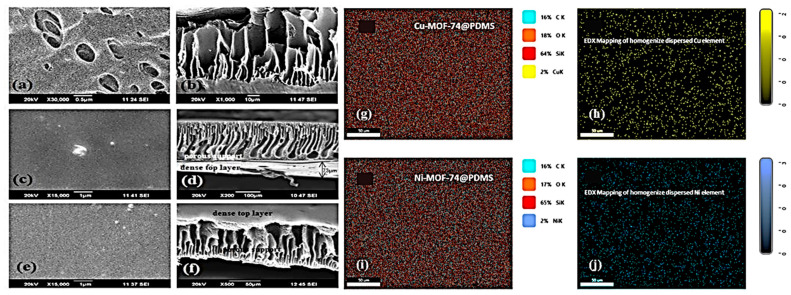
SEM images of (**a**) the surface of the PSF support, (**b**) the cross-section of the PSF support, (**c**) the surface of Cu-MOF-74@PDMS/PSF, (**d**) the cross-section of Cu-MOF-74@PDMS/PSF, (**e**) the surface of Ni-MOF-74@PDMS/PSF, and (**f**) the cross-section of Ni-MOF-74@PDMS/PSF, together with EDX elemental maps of selected (**g**,**h**) Cu-MOF-74@PDMS and (**i**,**j**) Ni-MOF-74@PDMS mixed-matrix membranes.

Scanning electron microscopy (JEOL Ltd., Tokyo, Japan) was used to evaluate the morphology of the MOF powders, pristine PDMS, the unsupported MOF-74@PDMS mixed-matrix membranes, the porous PSF support membrane, and the corresponding PDMS/PSF composite membranes, as shown in Figure 8, Figure 9, Figure 10 and Figure 11. The SEM images were recorded at magnifications appropriate to the structural features being examined. Higher magnifications were used to assess surface texture- and filler-related features, whereas lower magnifications were used for cross-sectional images to capture the membrane thickness and overall layer structure. Therefore, surface and cross-sectional images were interpreted within their respective comparison groups. The Cu-MOF-74 powder (Figure 8A,B) appears as faceted, plate-like crystallites, whereas the Ni-MOF-74 powder (Figure 8C,D) shows a less uniform and more aggregated morphology. In contrast, pristine PDMS (Figure 8E,F) exhibits a smooth surface and a dense, nonporous cross-section, consistent with the expected morphology of a neat PDMS film. These observations provide the morphological baseline for assessing the effect of MOF incorporation into the PDMS phase [18,46].

For the unsupported MOF-74@PDMS mixed-matrix membranes (Figure 9 and Figure 10), the membranes containing 0.5 and 1 wt.% filler show relatively smooth surfaces and continuous dense cross-sections at the examined magnifications, suggesting that the dispersed phase is reasonably well accommodated within the PDMS matrix at these loadings. Among these samples, the 1 wt.% Cu-MOF-74@PDMS membrane appears to show the most uniform surface morphology at the examined magnifications. When the filler loading is increased to 1.5 and 2 wt.%, the membrane surfaces become progressively rougher and more particle-rich regions are visible, while the cross-sections also become more heterogeneous, with more obvious protrusions and particle-associated irregularities. These features are consistent with increasing filler agglomeration at higher loading. Such non-uniform filler distribution is commonly associated with poorer polymer–filler interfacial compatibility and can adversely affect both separation performance and mechanical integrity, which is consistent with the gas-permeation trends observed in the present study [18,24,46].

The PSF support membrane (Figure 11a,b) exhibits an asymmetric porous structure. Its surface contains open pores, while the cross-section shows a porous substructure with elongated finger-like voids, which is characteristic of a phase-inversion-derived PSF support. After coating with the PDMS/MOF selective layer, the composite membranes (Figure 11c–f) exhibit a comparatively smooth, dense top surface over the porous PSF support. In the cross-sectional images, a distinct dense PDMS/MOF layer is visible above the porous support. The thickness value shown in Figure 11d represents a localized SEM cross-sectional measurement of the PDMS/MOF dense selective coating, while the average PDMS selective-layer thickness was approximately 60–80 μm, as reported in Section 3.1. The interface between the two layers appears continuous, with no obvious delamination at the examined magnifications. These observations indicate that the PDMS layer effectively covers the PSF support and serves as the principal gas-selective barrier, while the PSF substrate acts as the mechanical scaffold [25].

The EDX elemental maps shown in Figure 11g–j correspond to the Cu-MOF-74@PDMS and Ni-MOF-74@PDMS mixed-matrix membranes. In both cases, the Cu and Ni signals are detected across the mapped surface regions, supporting the presence of the MOF phase within the PDMS matrix and suggesting that the metal-containing filler is not confined to only a few isolated macroscopic domains in the analyzed areas. However, because EDX mapping at this scale is surface-sensitive and does not quantify filler dispersion through the full membrane thickness, these results should be interpreted as supportive rather than definitive evidence of homogeneous filler distribution. SEM and EDX observations indicate that low-to-moderate MOF loadings produce more uniform membrane morphologies, whereas higher loadings promote aggregation and structural heterogeneity. Accordingly, the 1 wt.% loading appears to provide the most favorable balance between filler dispersion, membrane integrity, and gas-separation performance [24,46].

Based on SEM observations, the MOF-74 particles used in this study are in the micrometer size range. Despite this relatively large particle size, acceptable dispersion was achieved at low filler loadings (≤1 wt.%), which contributed to the observed improvements in gas transport behavior.

### 4.4. X-Ray Diffraction (XRD) Analysis

**Figure 12:**
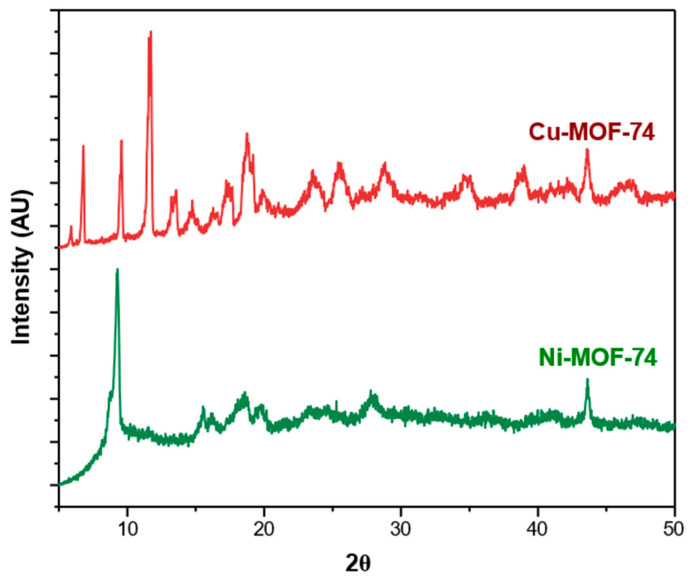
XRD spectra of pristine Cu-MOF-74 and Ni-MOF-74.

X-ray diffraction was used to evaluate the structural character of the MOF fillers and the polymeric membrane phases. As shown in Figure 12, both Cu-MOF-74 and Ni-MOF-74 exhibit distinct Bragg reflections, indicating that the synthesized fillers are crystalline. The Cu-MOF-74 pattern shows several well-resolved reflections over the scanned range, whereas the Ni-MOF-74 pattern is dominated by a strong low-angle reflection accompanied by weaker secondary peaks. This overall behavior is consistent with reported diffraction characteristics of MOF-74-type materials. The present patterns are best interpreted as evidence of crystalline MOF-74-type phases rather than over-assigned to specific crystallographic planes based on these scans alone, because the relative intensities and widths of MOF-74 reflections can vary with the metal center, synthesis route, activation state, and crystallite size.

**Figure 13:**
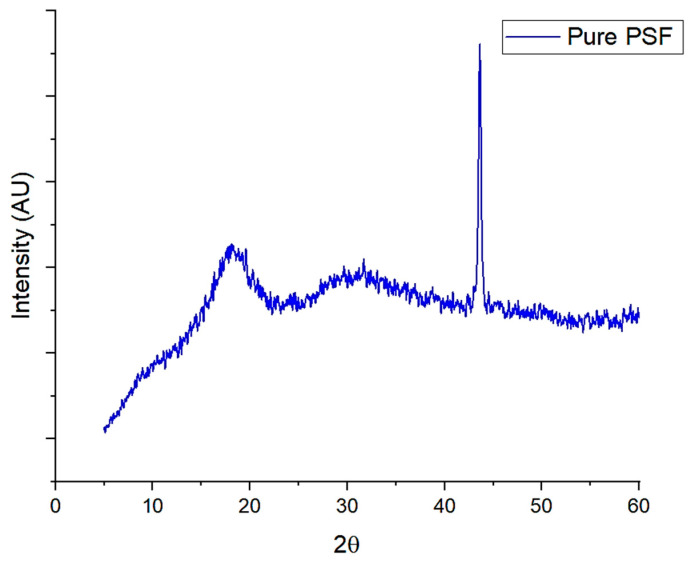
XRD spectra for pure PSF.

The XRD pattern of the PSF support membrane is shown in Figure 13. The diffractogram shows a broad diffuse halo centered approximately in the 18–20° region, indicating that the PSF support is predominantly amorphous. Although a sharp feature is also visible in the high-angle region near 43–45°, the overall diffractogram is still characterized mainly by diffuse scattering rather than multiple sharp crystalline reflections. Therefore, the PSF support is most appropriately described as a largely amorphous polymer matrix. This interpretation is consistent with the widely accepted amorphous character of polysulfone and with reports showing that PSF-based membrane patterns exhibit a broad reflection near 19°, together with a narrower feature near 45° [47].

**Figure 14:**
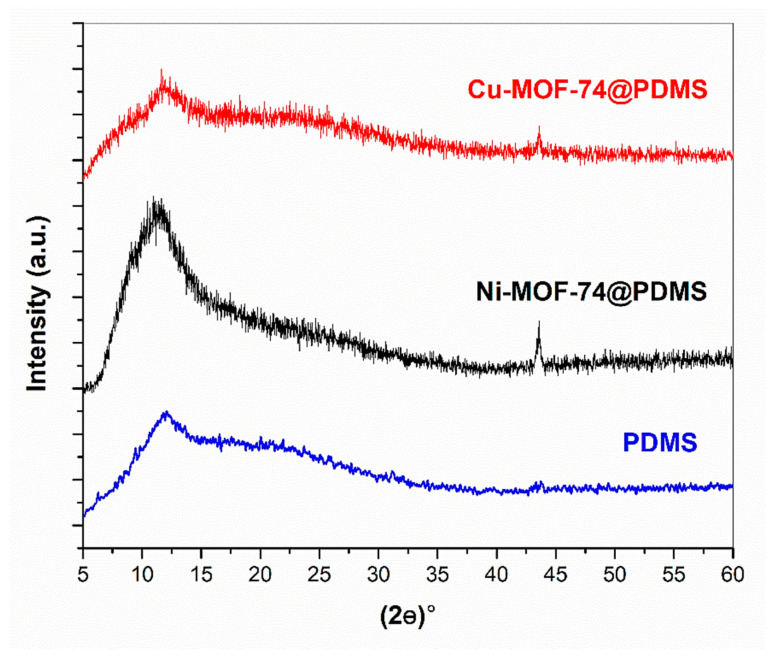
XRD spectra of pristine PDMS, Ni-MOF-74@PDMS, and Cu-MOF-74@PDMS.

Figure 14 compares pristine PDMS with the Cu-MOF-74@PDMS and Ni-MOF-74@PDMS mixed-matrix membranes. The pristine PDMS membrane exhibits a broad diffuse maximum in the low-angle region, around 10–15°, characteristic of an amorphous PDMS phase. After incorporation of Cu-MOF-74 or Ni-MOF-74, this broad halo remains the dominant feature in both mixed-matrix membranes, indicating that the continuous PDMS phase retains its essentially amorphous character after filler addition. A weak high-angle reflection is visible near 43–45° in the MOF-containing membranes, particularly in Ni-MOF-74@PDMS; however, because a small feature in this region is also present in pristine PDMS, this reflection should not be assigned solely to the dispersed MOF phase. Instead, the XRD results indicate that the filler’s crystalline contribution is weak and only partially resolved against the dominant amorphous polymer background, as expected at low MOF loading [46,48]. The SEM thickness value represents a localized measurement, while the average selective-layer thickness determined using a digital micrometer was approximately 60–80 μm.

The XRD results show that the Cu-MOF-74 and Ni-MOF-74 fillers are crystalline, whereas the PSF support and PDMS matrix are predominantly amorphous. In the MOF-filled PDMS membranes, the persistence of the broad amorphous polymer halo indicates that MOF incorporation does not fundamentally alter the structural state of the polymer matrix. At the same time, the mixed-matrix membrane patterns do not justify detailed indexing of individual MOF reflections within the composite films. Thus, the most defensible conclusion from the present XRD data is that crystalline MOF fillers were incorporated into an essentially amorphous polymer phase, while the diffraction response of the MMMs remains dominated by the polymer background [33,46,47,48,49].

### 4.5. Ultimate Tensile Strength Analysis

**Figure 15:**
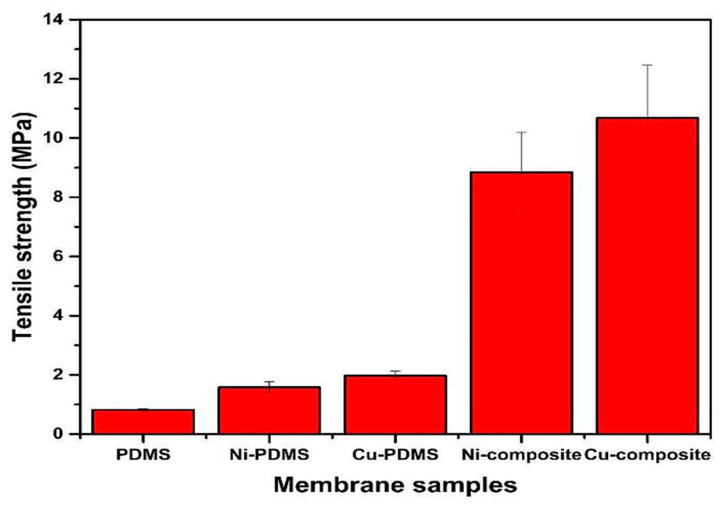
Ultimate tensile strength of pristine PDMS, PDMS-based mixed-matrix membranes, and PDMS/PSF composite membranes.

Ultimate tensile strength (UTS) measurements were carried out to evaluate the effects of MOF incorporation and PSF support-layer reinforcement on membrane mechanical integrity. As shown in Figure 15, pristine PDMS exhibited the lowest tensile strength, at 0.81 MPa. After incorporation of MOF-74 into the PDMS matrix, the tensile strength increased to 1.55 MPa for the Ni-PDMS membrane and 1.97 MPa for the Cu-PDMS membrane. This moderate increase suggests that the dispersed MOF phase can reinforce the soft PDMS matrix when the filler is reasonably well accommodated within the polymer phase. Such behavior is consistent with previous reports showing that mechanical enhancement in PDMS-based mixed-matrix membranes depends strongly on polymer–filler compatibility and effective stress transfer across the interface, while poor compatibility or aggregation can compromise membrane robustness [18,24,46].

A much larger increase in tensile strength was observed after fabrication of the dual-layer composite membranes on the PSF support. The Ni-composite membrane reached 8.84 MPa, while the Cu-composite membrane reached 10.68 MPa. Compared with pristine PDMS, these values correspond to approximately 10.9- and 13.2-fold increases, respectively. These results indicate that the major mechanical reinforcement arises from the composite architecture rather than from MOF addition alone. This interpretation is consistent with the established role of polysulfone as a mechanically robust membrane material and with the general behavior of thin-film composite membranes, in which the porous support layer provides most of the structural strength. In contrast, the dense top layer primarily contributes to separation performance [25,50,51].

Comparison of the unsupported MMMs and the supported composite membranes further indicates that MOF incorporation and support-layer reinforcement contribute at different levels. In the unsupported PDMS membranes, the increase from 0.81 MPa to 1.55–1.97 MPa suggests modest reinforcement of the polymer matrix by the rigid MOF phase. In contrast, once the same selective layer is integrated with the PSF support, the tensile strength increases dramatically to 8.84–10.68 MPa, indicating that the support layer bears a substantial fraction of the applied mechanical load. Within both membrane classes, the Cu-containing membrane exhibited slightly higher tensile strength than the corresponding Ni-containing membrane. In the present study, this trend is most reasonably interpreted as a morphology-related effect, consistent with the smoother and more uniform appearance of the Cu-containing membrane in the SEM analysis, rather than as proof of a fundamentally different reinforcement mechanism [18,24,46].

The UTS results are also consistent with the SEM observations. The cross-sectional SEM micrographs of the composite membranes show a distinct dense PDMS/MOF top layer deposited on the porous PSF support, together with a continuous interface and no obvious delamination at the examined magnifications. This structural continuity provides a reasonable explanation for the substantial improvement in tensile strength, as efficient adhesion between the selective layer and the support can facilitate stress distribution across the composite. Conversely, the rougher and more heterogeneous morphologies observed at higher filler loadings in the unsupported MMMs would be expected to generate stress-concentration sites and reduce effective load transfer [24,25,46].

The tensile data support two main conclusions. First, incorporation of MOF-74 into PDMS provides measurable, yet limited, reinforcement of the unsupported mixed-matrix membrane. Second, the PDMS/PSF composite configuration is responsible for the dominant enhancement in mechanical robustness, owing to the mechanically strong PSF scaffold and the intact dual-layer structure. This marked increase in tensile strength is important for practical gas-separation applications, since membrane handling, module fabrication, and resistance to deformation under operating pressure all depend strongly on mechanical stability [50,51]. Although the present work focused on single-gas permeation analysis to evaluate intrinsic membrane transport behavior, future studies should investigate mixed-gas separation performance under realistic flue-gas conditions.

## 5. Conclusions

In this study, dual-layer PDMS/PSF composite membranes incorporating Cu-MOF-74 and Ni-MOF-74 were fabricated and evaluated for CO_2_ separation. The results indicate that integrating MOF-74 fillers into the PDMS selective layer improved gas-separation performance under the present test conditions, while the PSF support provided substantial mechanical reinforcement to the membrane structure. Among the tested formulations, the membrane containing 1 wt.% Cu-MOF-74 exhibited the best separation performance, achieving a CO_2_ permeance of 912.5 GPU and an ideal CO_2_/N_2_ selectivity of 94.75. In comparison, the corresponding Ni-MOF-74-based membranes showed lower selectivity, indicating that the Cu-containing system provided more favorable CO_2_/N_2_ separation performance under the present test conditions.

Morphological and spectroscopic analyses supported the presence of the MOF phase in the PDMS matrix and the formation of an intact dual-layer membrane structure. SEM observations indicated that low-to-moderate filler loading produced more uniform membranes, whereas higher loadings promoted aggregation and structural heterogeneity. XRD confirmed the crystalline nature of the MOF fillers and the predominantly amorphous character of the polymer phases, while FTIR confirmed the presence of characteristic functional groups associated with PDMS, PSF, and MOF-74 in the fabricated membranes. Mechanical testing further indicated the advantage of the composite design: although MOF addition alone provided only modest reinforcement to PDMS, the PDMS/PSF configuration increased tensile strength markedly from 0.81 MPa for pristine PDMS to 10.68 MPa for the Cu-MOF-74/PDMS/PSF membrane.

It should be noted that long-term stability and durability under continuous operation were not investigated in the present study. Such evaluation is essential for practical deployment and will be considered in future work, particularly under conditions involving mixed gases, humidity, and extended operating durations. These findings indicate that the dual-layer PDMS/PSF architecture is a promising strategy for simultaneously improving CO_2_ separation performance and mechanical robustness. The optimized Cu-MOF-74-containing membrane therefore shows strong potential for membrane-based carbon capture applications. Since the present study is limited to single-gas permeation measurements, future work should include mixed-gas CO_2_/N_2_ testing, as well as long-term stability evaluation under humid and impurity-containing feed conditions, to assess the practical applicability of these membranes in more realistic operating environments.

## Figures and Tables

**Table 1:** Composition of the prepared PSF support, PDMS mixed-matrix, and PDMS/PSF composite membranes.

Membrane/Sample	Membrane Type	PSF Support Composition	PDMS Layer Composition	MOF-74 Loading
PSF support	Porous support membrane	PSF/NMP solutions containing 10, 20, and 25% (*w*/*v*) PSF, prepared by NIPS in deionized water	—	—
Pristine PDMS	Unsupported dense PDMS membrane	—	4 g PDMS in 10 mL toluene; 40% (*w*/*v*); elastomer:curing agent = 10:1	0 wt.%
Cu-MOF-74@PDMS	Unsupported Cu-MOF-74/PDMS mixed-matrix membrane	—	4 g PDMS in 10 mL toluene; 40% (*w*/*v*); elastomer:curing agent = 10:1	0.5, 1.0, 1.5, and 2.0 wt.% Cu-MOF-74 relative to PDMS
Ni-MOF-74@PDMS	Unsupported Ni-MOF-74/PDMS mixed-matrix membrane	—	4 g PDMS in 10 mL toluene; 40% (*w*/*v*); elastomer:curing agent = 10:1	0.5, 1.0, 1.5, and 2.0 wt.% Ni-MOF-74 relative to PDMS
PDMS/PSF	Pristine dual-layer composite membrane	Porous PSF support prepared by NIPS	4 g PDMS in 10 mL toluene; 40% (*w*/*v*); elastomer:curing agent = 10:1	0 wt.%
Cu-MOF-74@PDMS/PSF	Cu-MOF-74-containing dual-layer composite membrane	Porous PSF support prepared by NIPS	4 g PDMS in 10 mL toluene; 40% (*w*/*v*); elastomer:curing agent = 10:1	0.5 and 1.0 wt.% Cu-MOF-74 relative to PDMS
Ni-MOF-74@PDMS/PSF	Ni-MOF-74-containing dual-layer composite membrane	Porous PSF support prepared by NIPS	4 g PDMS in 10 mL toluene; 40% (*w*/*v*); elastomer:curing agent = 10:1	0.5 and 1.0 wt.% Ni-MOF-74 relative to PDMS

**Table 2:** Benchmark comparison of the optimized 1 wt.% Cu-MOF-74@PDMS/PSF membrane with reported CO_2_/N_2_ separation membranes.

Membrane/Sample	CO_2_ Permeance (GPU)	CO_2_/N_2_ Selectivity	Temperature	Pressure	Ref.
1 wt.% Cu-MOF-74@PDMS/PSF dual-layer composite membrane	**912.5**	**94.75**	**25 °C**	**5 bar**	This work
PSf/PDMS-plasma/Pebax-TPP 50 wt.% composite membrane	>400 GPU	~28	35 °C	7 bar	[37]
Pebax/PDMS/PES hollow fiber membrane	1253	34.91	25 °C	1 bar	[38]
Pebax/PDMS/PAN composite hollow fiber membrane	~480	42.0	25 °C	2 bar	[39]
Pebax-aGO/PDMS/PSf mixed-matrix composite membrane	208.9	40	35 °C	7 bar	[25]
Pebax-2533/ZIF-7 25 wt.%–PSf composite membrane	21.5	50.4	25 °C	3.04 bar	[40]
PDMS/PSf composite membrane	21.5	43.88	25 °C	10 bar	[41]
PIM-1@MOF thin-film composite membrane	5018	31	35 °C	1.0 bar gauge	[42]
PEG/PDMS&MOF/PAN TFC membrane	1990	39	35 °C	1 bar	[27]
PBE/MOF-808 40% TFC-MMM	1069	52.7	30 °C	1 bar	[43]

The reported membrane performances were collected from the literature and measured under different operating conditions; therefore, a direct quantitative comparison should be interpreted cautiously.

## Data Availability

The data supporting the findings of this study are available from the corresponding author upon reasonable request.

## Supplementary Materials

The following supporting information can be downloaded at: https://www.mdpi.com/article/10.3390/polym18111303/s1, Figure S1: FTIR spectra of Cu-MOF-74 and Ni-MOF-74 powders.; Figure S2: FTIR spectra of pristine PDMS, Cu-MOF-74@PDMS, and Ni-MOF-74@PDMS mixed-matrix membranes.

## Nomenclature

MMM	Mixed-Matrix Membrane	%	Percentage
MOF	Metal Organic Framework	PU	Polyurethane
PDMS	Polydimethylsiloxane	NCs	Nanocrystals
PSF	Polysulfone	CA	Cellulose Acetate
PC	Polycarbonate	CTA	Cellulose Triacetate
PES	Polyethersulfone	PEI	Polyetherimide
PI	Polyimide	PPO	Polyphenylene oxide
Ni	Nickel	vol.	Volume
Cu	Copper	Fig	Figure
CO_2_	Carbon Dioxide	µm	Micrometer
O_2_	Oxygen	cm	Centimeter
N_2_	Nitrogen	kV	Kilovolt
SEM	Scanning Electron Microscopy	°	Degree
XRD	X-ray Diffraction	kW	Kilowatt
EDX	Energy Dispersive X-Ray	mA	Milliampere
UTS	Ultimate Tensile Strength	mm	Millimeter
FT-IR	Fourier-transform infrared spectroscopy	min	Minute
MWCNT	Multi-Walled Carbon Nanotubes	kN	Kilo Newton
ASTM	American Society for Testing and Materials	Wt.	Weight
P	Permeability	°C	Degree Celsius
IPCC	Intergovernmental Panel on Climate Change	H	Hydrogen
Si	Silicon	∆	Differential
CH_3_	Methyl group	L	Thickness
Si(CH_3_)_2_O	Repeating unit of polydimethylsiloxane	A	Area
FFV	Fractional Free Volume	p	Pressure
ZIF	Zeolitic imidazolate frameworks	α	Ideal Selectivity
Mg	Magnesium	nm	Nanometer
Co	Cobalt	C	Carbon
Mn	Manganese	-O	Oxygen Bond
OH^-^	Hydroxyl	>	Greater Than
Q	Volumetric Gas Flow Rate	MPa	Mega Pascal
