# Supplementary material for: Dual-Layer PDMS/Polysulfone Composite Membranes Incorporating Cu-MOF-74 for Enhanced CO2 Capture Performance
Source: Polymers (Basel). 2026 May 26;18(11):1303. doi: 10.3390/polym18111303 (PMC13259534; doi:10.3390/polym18111303)
Supplement: Supplementary file 1 [file polymers-18-01303-s001.zip › polymers-4294358-supplementary.pdf]

Article

Dual-Layer PDMS/Polysulfone Composite Membranes Incorporating Cu-MOF-74 for Enhanced CO2 Capture Performance

Shoaib Ahsan, Muhammad Ahsan *, Tayyaba Noor, Sarah Farrukh and Subhan Ali

School of Chemical and Materials Engineering (SCME), National University of Sciences and Technology (NUST), Sector H-12, Islamabad 44000, Pakistan; sahsan.phdscme@student.nust.edu.pk (S.A.); tayyaba.noor@scme.nust.edu.pk (T.N.); sarah.farrukh@scme.nust.edu.pk (S.F.); subhan.che8scme@student.nust.edu.pk (S.A.)

* *Correspondence: ahsan@scme.nust.edu.pk*

S1. FTIR Spectra of MOF-74 Powders and PDMS-Based Mixed-Matrix Membranes

S1.1. FTIR analysis of Cu-MOF-74 and Ni-MOF-74 powders

The FTIR spectra of Cu-MOF-74 and Ni-MOF-74 powders are shown in Figure S1. Both spectra exhibit the characteristic absorption features of MOF-74-type frameworks. The broad absorption in the 3600–3200 cm^{-1} region is assigned to O–H stretching vibrations from hydroxyl groups and/or adsorbed moisture associated with the MOF-74 structure [1]. The bands in the 1600–1400 cm^{-1} region correspond to asymmetric and symmetric stretching vibrations of coordinated carboxylate groups from the organic linker [1]. Additional absorptions in the lower-wavenumber region are attributed to C–O, aromatic ring, and metal–oxygen framework vibrations [1]. These bands are consistent with the expected FTIR profile of Cu-MOF-74 and Ni-MOF-74 powders.

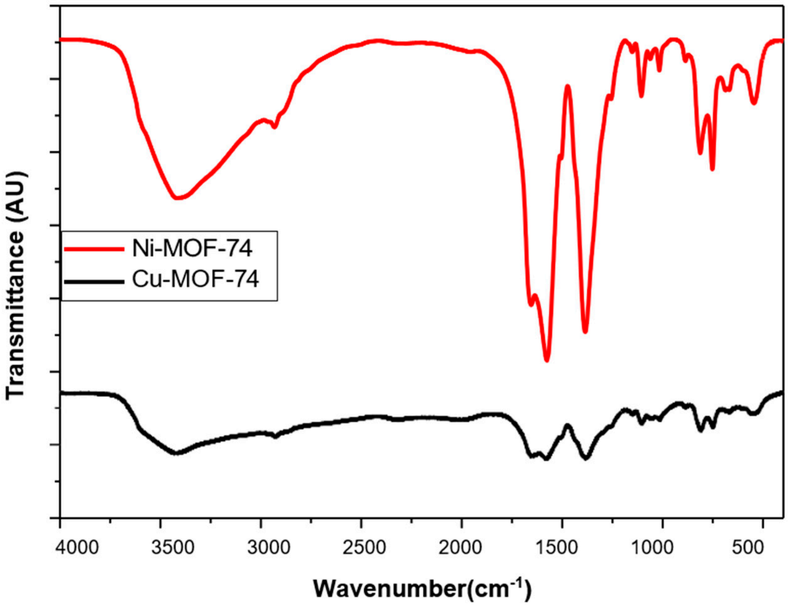

Figure S1. FTIR spectra of Cu-MOF-74 and Ni-MOF-74 powders.

S1.2. FTIR analysis of pristine PDMS and MOF-74@PDMS mixed-matrix membranes

Figure S2 shows the FTIR spectra of pristine PDMS, Cu-MOF-74@PDMS, and Ni-MOF-74@PDMS mixed-matrix membranes. Pristine PDMS shows the characteristic absorption bands of the siloxane network. The bands near 2960–2905 cm^{-1} are assigned to C–H stretching vibrations of Si–CH_3 groups, while the band near 1260 cm^{-1} corresponds to Si–CH_3 deformation [2]. The strong absorption envelope in the 1090–1020 cm^{-1}

region is attributed to Si–O–Si stretching of the PDMS backbone. The band near 800 cm^{-1} is assigned to Si–CH_3 rocking and Si–C-related vibration [2].

The Cu-MOF-74@PDMS and Ni-MOF-74@PDMS spectra retain the main PDMS absorption bands, indicating that the PDMS backbone remained chemically stable after MOF incorporation. Weak MOF-related contributions may occur in the O–H stretching region and in the 1600–1400 cm^{-1} carboxylate region. However, these bands are not strongly separated because the filler loading is low and the PDMS absorptions dominate the spectra. This behavior is consistent with Cu/Ni-MOF-74-loaded PDMS mixed-matrix membranes, where weak filler-related bands may be masked by the polymer matrix at low loading [3].

The absence of major new absorption bands or large shifts in the PDMS characteristic peaks suggests that the MOF-74 fillers were physically incorporated into the PDMS matrix rather than forming new covalent bonds with the polymer phase. Therefore, Figure S2 supports the retention of the PDMS siloxane structure after filler addition and provides reference spectra for interpreting the final PDMS/PSF composite membranes discussed in the main text.

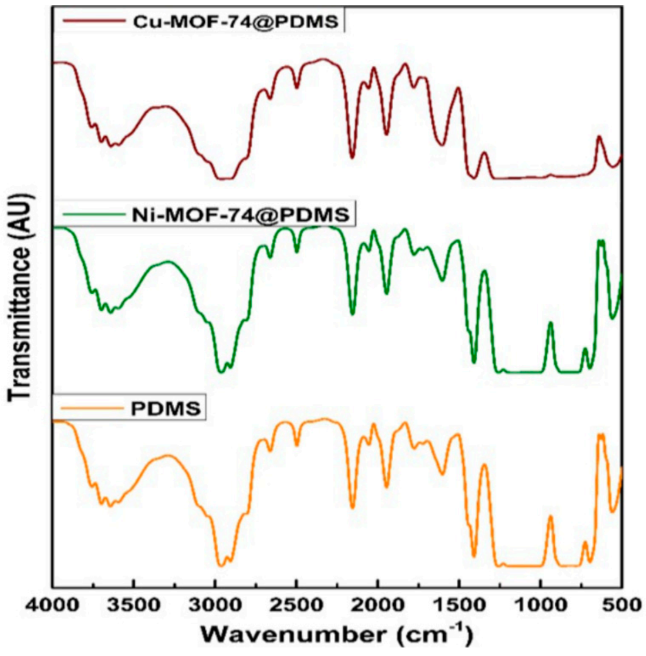

Figure S2. FTIR spectra of pristine PDMS and Cu-MOF-74@PDMS and Ni-MOF-74@PDMS mixed-matrix membranes.

References

1. Xiao, T.; Liu, D. The Most Advanced Synthesis and a Wide Range of Applications of MOF-74 and Its Derivatives. *Microporous and Mesoporous Materials* **2019**, *283*, 88–103, doi:10.1016/J.MICROMESO.2019.03.002.
2. Goel, V.; Tanwar, R.; Saikia, A.K.; Mandal, U.K. Separation Characteristics of Surface Modified Polysulfone Ultrafiltration Membrane Using Oxidative Catalytic Polymerization of Aniline. *Journal of Polymer Materials* **2024**, *39*, 283–305, doi:10.32381/JPM.2022.39.3-4.8.
3. Ali, S.; Farrukh, S.; Karim, S.S.; Noor, T.; Liaquat, S.; Sultan, A. Investigation of the Effect of Ni and Cu Variant MOF-74 in the Polydimethylsiloxane (PDMS)-Based Mixed Matrix Membranes (MMMs) for Efficient Gas Separation Applications. *Environmental Science and Pollution Research* **2023**, *30*, 109453–109468, doi:10.1007/S11356-023-30029-2.
